# Porcine Forebrain Vacuolization Associated with Wasting in Pigs: A Novel Pathological Outcome Associated with Vitamin–Mineral Deficiency?

**DOI:** 10.3390/ani13142255

**Published:** 2023-07-10

**Authors:** E. Ruiz-Riera, E. Vidal, A. Canturri, A. Lehmbecker, M. Cuvertoret, C. Lopez-Figueroa, W. Baumgärtner, M. Domingo, J. Segalés

**Affiliations:** 1 Departament de Sanitat i Anatomia Animals, Facultat de Veterinària, Campus de la Universitat Autònoma de Barcelona (UAB), 08193 Bellaterra, Spain; elisa.ruizriera@gmail.com (E.R.-R.); cantu028@umn.edu (A.C.); mcuvertoret@gmail.com (M.C.); carlos.lopez@irta.cat (C.L.-F.); mariano.domingo@uab.cat (M.D.); 2 Unitat Mixta d’Investigació IRTA-UAB en Sanitat Animal, Centre de Recerca en Sanitat Animal (CReSA), Campus de la Universitat Autònoma de Barcelona (UAB), 08193 Bellaterra, Spain; 3 IRTA, Programa de Sanitat Animal, Centre de Recerca en Sanitat Animal (CReSA), Campus de la Universitat Autònoma de Barcelona (UAB), 08193 Bellaterra, Spain; 4 OIE Collaborating Centre for the Research and Control of Emerging and Re-Emerging Swine Diseases in Europe (IRTA-CReSA), 08193 Bellaterra, Spain; 5 Department of Veterinary Population Medicine, College of Veterinary Medicine, University of Minnesota, St. Paul, MN 55455, USA; 6 Veterinary Diagnostic Laboratory, College of Veterinary Medicine, University of Minnesota, St. Paul, MN 55455, USA; 7 Department of Pathology, University of Veterinary Medicine, 30545 Hannover, Germany; annika-lehmbecker@idexx.com (A.L.); wolfgang.baumgaertner@tiho-hannover.de (W.B.)

**Keywords:** sus scrofa, brain vacuolization, spongiosis, wasting, metabolic disorder

## Abstract

**Simple Summary:**

The term wasting is a clinical name describing a physical condition characterized by growth retardation, usually of multifactorial origin. The present study describes an apparently new condition of pigs characterized clinically by wasting and pathologically by vacuolization of the brain. During 2016–2018, animals from eight farms were weaned in good body condition, and after 1–2 weeks, they started losing weight. To investigate potential causes of this condition, apparently sick and healthy pigs from each herd of eight affected farms were studied by means of histopathology, transmission electron microscopy and detection of usual infectious agents. Histopathologically, the most consistent lesion was neuropil vacuolization of the prosencephalon, mainly located in the thalamic nuclei and in the transition between the white and grey matter of the neocortex. Electron microscopy of some of these sick animals showed preserved axons, with dilated myelin sheaths (interpreted as edema of myelin sheath). The literature suggests this lesion type is linked to congenital or metabolic (toxic/deficiency) scenarios. The present case was probably a vitamin/mineral deficiency since supplementation with nutritional complexes solved the problem.

**Abstract:**

The term wasting refers to a clinical sign used to describe a physical condition characterized by growth retardation, usually of multifactorial origin. The objective of the present study was to describe for the first time a pathological process characterized by forebrain neuropil vacuolization in pigs showing wasting without conspicuous neurological signs. To characterize the lesions pathologically, affected and non-affected pigs from eight of these farms were investigated. Histologically, the most consistent lesion was neuropil vacuolization of the prosencephalon, mainly located in the thalamic nuclei and in the transition between the white and grey matter of the neocortex (40/56 in sick and 4/30 in healthy pigs). In the most severe cases, the vacuolation also involved the midbrain, cerebellar nuclei and, to a lesser extent, the medulla oblongata. Vacuolization of the forebrain was associated with pigs experiencing marked emaciation and growth retardation. Although the specific cause of the present case remained unknown, the preventive use of multivitamin and mineral complexes in drinking water ameliorated the condition, strongly suggesting a metabolic origin of the observed condition.

## 1. Introduction

A wasting disease is characterized by the gradual deterioration of an individual, usually with loss of strength and muscle mass, which may be accompanied by loss of appetite, and is a sign of chronic disease [1]. Another synonym for wasting is cachexia, which refers to a loss in body mass and worsens the course of multiple underlying disease processes [2].

In animal production, growth retardation of animals is a concern due to economic losses and animal welfare. In swine, there are many causes of wasting, including social and environmental stressors, which include nutritional factors, as well as infectious diseases. There are multiple risk factors that can lead to postweaning wasting, which are animal-related (litter, birth or weaning weight), facilities, management practices, vaccination, treatments and nutrition. Those can lead, under different circumstances, to starvation, malnutrition, dehydration and gastric ulceration which alter the growth of piglets [2,3]. In addition, many infectious diseases have also been described as causing wasting after weaning, such as Porcine circovirus 2 (PCV-2) systemic disease, porcine reproductive and respiratory syndrome (PRRS), enzootic pneumonia and swine dysentery, among many others [3,4].

Spongy state or spongiform degeneration is a neuropathological change that is often characterized by bilateral, symmetrical vacuolation more usually found in white than grey matter, and it is described in several animal diseases [5]. The vacuoles are sharply defined and vary in size; in severe cases, vacuoles may coalesce. It is caused by brain edema, loss of axons or myelin, and vacuolation of neurons, glial cells or their processes, and it is also a common alteration related to age [6]. Lesions can be widespread in the central nervous system but may target specific areas, and the peripheral nervous system can also be affected [5]. Spongiform degeneration of the white matter in animals can result from ammonia poisoning, hexachlorophene intoxication, closantel intoxication and poisoning with bromethalin-derived rodenticides [5]. Hepatic encephalopathies secondary to infectious, metabolic and degenerative liver diseases can also manifest with different degrees of white matter vacuolation. Other causes such as congenital or breed-related brain vacuolization have been described, mostly in dogs and cats. In pigs, congenital tremor is the most important cause of white matter vacuolization in the brain. This condition affects newborn piglets and can last for several weeks. Lesions are restricted to the central nervous system, with histological changes consisting in hypomyelination of the white matter of the spinal cord and brain stem [7,8]. Infectious and toxic agents, as well as hereditary conditions account for the specific causes of congenital tremors [7]. Regarding spongiform changes affecting grey matter, prion diseases are the most relevant ones. However, there is no evidence for naturally occurring transmissible spongiform encephalopathies in swine. There are scarce references regarding spongiosis and neuropil vacuolization affecting swine, exclusively related to the experimental induction of spongiform encephalopathies in swine brains [9]. One study indicated that histopathological examination of brains from healthy pigs showed localized vacuolar changes, predominantly in the rostral colliculus, which were similar to the neuropil vacuolation featured in the transmissible spongiform encephalopathies; however, this study concluded that such vacuolization was not caused by a transmissible agent and was probably an insignificant change [10,11].

The objective of the present study was to characterize the histological lesions of brain vacuolization observed in pigs displaying a wasting condition not apparently associated with infectious diseases of swine. These lesions were characterized by describing their specific location, distribution and severity, as well as by immunohistochemical and transmission electron microscopy methods. In addition, several infectious and non-infectious diseases were ruled out by clinical assessment and laboratory analyses.

## 2. Materials and Methods

### 2.1. Clinical Presentation 

A swine integrator company located in Spain experienced a significant problem in 61 out of their 136 herds that lasted variably among herds between August 2016 and March 2018. The duration of the disorder ranged from around 1.5 years in those farms affected for the longest period and about 1–2 months in the least impacted ones. Clinically affected pigs were characterized by anorexia with no fever and loss of body condition starting at 1–2 weeks post-weaning (around 4–6 weeks of life). Animals showed progressive wasting with no evidence of respiratory, digestive, or neurological signs, with the most evident weight loss seen around 6–8 weeks of age. The condition affected between 5 and 25% of the piglets depending on the batch and farm, and in most of the cases they had to be euthanized due to animal welfare issues; the overall direct mortality associated with the disorder was rather low (lower than 4% of the affected animals). Compared to non-affected pen-mates, affected animals weighed between 0.5 and 6 kg less by the end of the nursery period. All farms applied a vaccination schedule against *Aujeszky’s diseases virus*, *Porcine Parvovirus*, *Erysipelothrix rhusiopathiae*, *Escherichia coli* and *Clostridium perfringens* type C in gilts and sows, and PCV-2 and, variably, *Porcine reproductive and respiratory syndrome virus* and *Mycoplasma hyopneumoniae* in weaned piglets.

The clinical disorder started remitting by early 2018 when a multivitamin and mineral complex-based treatment (Supplementary File S1) was implemented in a blanket in the drinking water during the starter feeding period (for a period of 7–10 days starting at 4 weeks of age) of the animals. The complex was administered until clinical signs remitted in each farm. From mid-2018 onwards, the condition was not further observed clinically.

### 2.2. Study Population and Data Collection

Eight different affected farms (Nos. 1 to 8) from the integration company were investigated (in one farm the study was conducted twice) by means of a follow-up study. First, a total of 100 piglets (males and females) per farm were ear-tagged at weaning (around 3–4 weeks of age) and clinically monitored by the responsible veterinarian of the farm until they started to show evidence of wasting. The weights from farms 1 to 5 and the first batch from farm 8 were noted (Table 1 and Supplementary File S2). Pigs were bled at three weeks of age and then every two weeks until disease presentation (blood was centrifuged at each time point, and serum was stored at −80 °C for further use). When clinical signs were fully present, mainly characterized by wasting and lack of respiratory, digestive and neurological signs (around 6–8 weeks of age), five emaciated and five apparently healthy pigs from each batch were submitted for diagnostic investigations (Supplementary File S3). From one farm (No. 7), only wasted animals were submitted; from the farm in which the study was conducted twice (No. 8), no apparently healthy pigs were received from the second monitored batch, consisting only of 5 clinically affected animals, with an additional group of 5 neonates with no clinical issues.

From three of the studied farms (Nos. 5, 6 and 7), a total of 17 pigs that were identified as clinically diseased during the peak of the problem were further monitored and submitted for necropsy and histopathological analysis one month later.

### 2.3. Sample Collection and Histopathology 

A standardized complete necropsy procedure was performed in all clinically affected and non-affected animals from farms 1 to 5 and the first batch of farm 8 at the peak of the clinical problem as well as in affected animals one month after the acute phase. Necropsies were conducted at the Servei de Diagnòstic de Patologia Veterinària (SDPV) from the *Universitat Autònoma de Barcelona* (UAB), Catalonia (Spain). Samples collected for routine histopathology from different tissues were fixed by immersion in 10% neutered buffered formalin. The following tissues were collected: nasal turbinates, lung, heart, tonsil, thymus, lymph nodes (tracheo-bronchial, mesenteric, and superficial inguinal), liver, spleen, kidney, stomach, small and large intestine, skeletal muscle and whole brain. From two farms (Nos. 6 and 7) and the second batch of farm 8, a necropsy was performed by field veterinarians, and the brain (including from the 5 neonates of farm 8) was taken and fixed by immersion in 10% neutered buffered formalin. Obtained tissues were embedded in paraffin, cut into 4 µm sections, stained with hematoxylin and eosin and examined under a light microscope.

Histopathological examination was conducted in a blinded fashion for the clinical status of the animals by a European College of Veterinary Pathologists-certified specialist.

### 2.4. Biochemical and Hematological Studies 

Biochemical and hematological studies (Supplementary Files S4 and S5) were performed in all pigs from two farms (farms 1 and 2), where the following parameters were studied: red blood cell (×10^6^ cells/μL) and leukocyte (×10^3^ cells/μL) counts, hemoglobin (g/dL), hematocrit (Ht, %), percentage of neutrophils, lymphocytes, monocytes and eosinophils, platelet number and total protein (g/dL). Also, some biochemical parameters of serum samples, namely alkaline phosphatase (U/L), calcium (mg/dL), iron (μg/dL), phosphorus (mg/dL) and magnesium (mg/dL), were evaluated. 

### 2.5. Determination of Heavy Metals

A wide panel of inorganic elements (Supplementary File S6) was evaluated in the serum of all animals from farms 1 to 4 (10 animals from each farm, five apparently healthy and five clinically diseased ones). These metals were determined by inductively coupled plasma mass spectrometry (ICP-MS), Agilent 7500ce. Serum samples were diluted within a solution of 0.05% (*w*/*v*) EDTA and 0.5% (*v*/*v*) NH_3_. The semiquantitative estimation was performed with the ICP-MS based on the curve of molar response versus atomic weight.

### 2.6. Histochemical and Immunohistochemical Studies

Several complementary tests were performed on tissues of both apparently healthy and clinically sick animals. Immunohistochemistry to detect PRRSV and PCV-2 antigens was performed in all necropsied animals having lung and lymph nodes as available material (60 animals in total). 

To neuropathologically characterize the brain lesions observed histopathologically, 14 selected animals with different severity of lesions were further studied, including four animals with no evidence of microscopic brain findings. In these animals, further immunohistochemical stains (Supplementary File S7) were performed to identify different cell markers in brain tissues including glial fibrillary acidic protein (GFAP) for astrocytes, prion protein (PrP^res^), parvalbumin (calcium-binding protein expressed at GABAergic inhibitory neuronal populations), neurofilaments 200 KDa for neurons and IBA1 (ionized calcium-binding adapter molecule 1) for microglial visualization. In addition, Luxol fast blue staining was used to evaluate the integrity and staining intensity of myelin fibers in the brain tissues of these 14 animals. 

Different anatomical areas were assessed to determine the distribution and intensity of the brain vacuolation, including frontal, temporal, occipital and parietal cortex, basal nuclei (caudate, putamen and pallidum), internal capsule, thalamus, hypothalamus, hippocampus, mesencephalon, medulla oblongata, cerebellar cortex and cerebellar nuclei. Severity was measured from 0 to 3, with 0 being non-affected and 3 given to areas with the maximum lesion intensity. 

### 2.7. Molecular Biology

Additional studies were performed on serum samples to detect common swine infectious agents able to cause wasting (Supplementary File S3), including PCR for detection of *Porcine circovirus 3* (PCV-3) [12], PCR for *Mycoplasma* spp. (to rule out eventual *M. suis* infection) and RT-PCR for the detection of PRRSV [13].

### 2.8. Transmission Electron Microscopy 

Formalin-fixed samples of two sick animals and one unaffected animal were transferred into 5% glutaraldehyde/cacodylate buffer and post-fixed for a minimum of 24 h. After incubation in 1% osmium acid, samples were transferred into an ascending alcohol series from 30% to finally 100% alcohol. Afterward, samples were embedded in epoxide. Semithin sections were used to determine regions of interest for ultrathin sections. Ultrathin sections were mounted on copper grids and treated with uranyl acetate and lead citrate. The evaluation was performed using a Zeiss EM 10C electron microscope [14].

### 2.9. Statistical Analyses

The data obtained from hematological, biochemical and heavy metal determinations as well as main gross and histological findings were subjected to statistical analysis. Firstly, the homoscedasticity of the variance of data was assessed using the Levene test; then, the *t*-test was used to verify if there were significant differences between the means of the hematological, biochemical and heavy metal values among animal groups. Next, we created 2 × 2 tables for each of the gross and histological findings to examine possible differences between piglet conditions using a chi-square test. Finally, to compare the weight of both normal and wasted piglets, a Wilcoxon–Mann–Whitney test was performed. The statistical significance level for the selected variables was set at *p*-value ≤ 0.05. The data analysis was performed using the R software version 4.0.2 (R Development Core Team, 2022).

## 3. Results

### 3.1. Clinical and Gross Findings

Affected animals submitted to necropsy suffered from stunted growth and poor body condition (Supplementary File S2) and had poor hair coat quality. Healthy animals did not show apparent clinical signs; however, those from farm 3 had a similar weight (7.4 kg vs. 9.6 kg) and body condition compared to the selected sick animals, so they were all considered as clinically affected (Supplementary File S2). Clinically normal pigs were significantly heavier than ill ones (*p* < 0.05). Different gross findings were noted, but erosions, ulceration of the *pars oesophagea* of the stomach and multifocal cranioventral pulmonary consolidation (suppurative bronchopneumonia) limited to apical and middle lobes were the most common ones found in both clinically healthy and affected animals (Table 1); however, no differences in frequency of gross lesions were observed between groups (*p* > 0.05). 

### 3.2. Biochemistry, Hematology and Heavy Metal Determination in Serum

No significant (*p* > 0.05) biochemical disturbances (Supplementary File S4) were found neither in healthy or sick animals (alkaline phosphatase, calcium, iron, phosphorus, magnesium). Values obtained were overall considered within the normal ranges for nursery pigs [15]. 

Regarding hematological parameters (Supplementary File S5), no significant differences were observed between both groups (*p* > 0.05). Two animals had some parameters related to red blood cells (red blood cell count, hemoglobin and hematocrit) below reference values [16], indicative of anemia; these animals showed gastric ulceration of the *pars oesophagea*. No significant statistical results were obtained.

Heavy metals (Supplementary File S6) selenium (Se), iodine (I), barium (Ba) and cerium (Ce) were significantly different when comparing studied groups (*p* < 0.05). Se, Ba and Ce concentrations were higher in wasted piglets, while I was higher in healthy pigs. Despite the difference in Se and I serum concentrations, both values were considered within normal range values [17,18]. No data on Ba and Ce concentrations were found in the literature. The remaining determined heavy metals were not significantly different in terms of serum concentration among studied groups (*p* > 0.05). 

### 3.3. Pathogen Detection

Only 3 out of 20 pigs tested (from farms 1 and 2) were positive for PCV-3 by PCR in serum; two of the animals were sick (from farm 2), while the third animal was healthy (farm 1). All tested animals were negative for RT-PCR (in serum) and immunohistochemistry (in lung tissue) for PRRSV, for immunohistochemistry for PCV-2 (in lymphoid tissues) as well as for *Mycoplasma* spp. PCR (in serum).

### 3.4. General Histopathological Findings

A wide range of histological lesions were observed (Table 1) The most consistent lesion was neuropil vacuolization of the prosencephalon, mainly located in the thalamic nuclei, mesencephalon and transition between the white and grey matter of the neocortex (40/56 in sick and 4/30 in healthy pigs). Other observed microscopic lesions were rather inconsistent and unspecific, such as catarrhal rhinitis and colitis, catarrhal or ulcerative gastritis, and suppurative bronchopneumonia; although these lesions were present in both groups of animals, they were slightly more prevalent in diseased pigs. A few animals from a couple of farms (Nos. 1 and 2) had inclusion body rhinitis, irrespectively of the health status of the animals. Brain vacuolation and non-suppurative gastritis were found in a significantly higher frequency in sick compared to healthy pigs (*p* < 0.05); the differences in frequency of the rest of the histopathological findings were not significant.

The 17 diseased pigs at the peak of the clinical problem that were followed up for an additional month (from farms 5, 6 and 7) did not show microscopic brain lesions. The neonate brains from farm 8 (submitted together with the second batch samples) did not have histopathological findings either.

### 3.5. Neuropathological Characterization 

**Figure 1:**
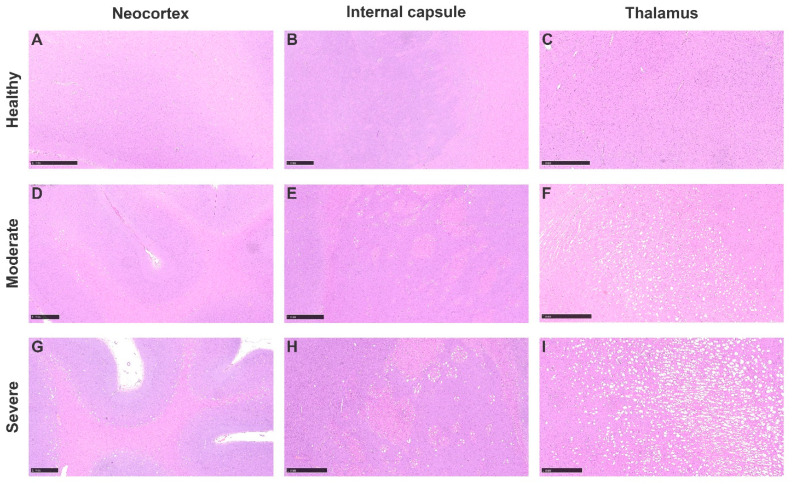
Brain vacuolation. Hematoxylin–eosin. Healthy animals with no vacuoles are shown in (**A**) neocortex, (**B**) internal capsule and (**C**) thalamus. Moderately affected cases are shown in (**D**) neocortex, (**E**) internal capsule and (**F**) thalamus. Note severe vacuolization cases in (**G**) neocortex and (**H**) internal capsule and severe affectation of (**I**) thalamus. All bar scales represent 1 mm.

Amongst all the submitted cases, 14 of them were selected based on histological findings to further characterize the histological brain lesions. Microscopic changes consisted of bilateral and symmetrical vacuolation of neuroparenchyma affecting different structures and in variable severity amongst different cases. Vacuoles were variable in size and mainly located in the thalamic nuclei, the mesencephalon and in the transition between the white and grey matter of the neocortex (Figure 1).

**Figure 2:**
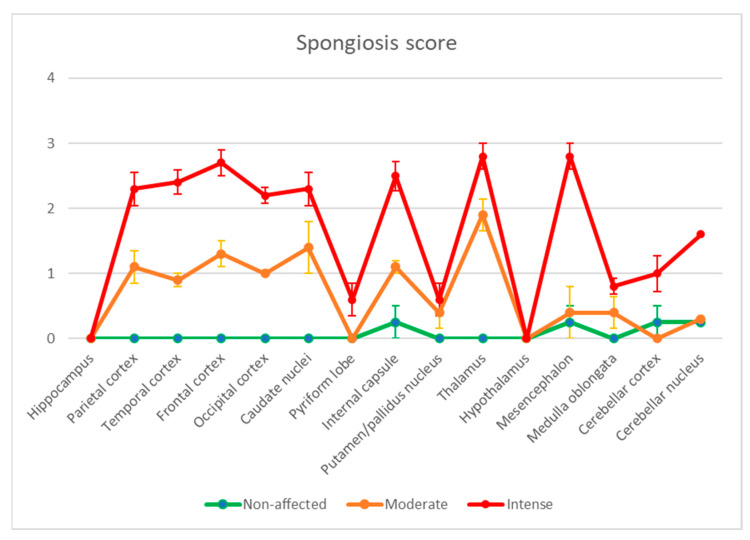
Brain distribution of the spongiform lesions. A semiquantitative score was given to the different brain areas studied from 0 (no lesion) to 3 (maximum intense vacuolation). The animals were grouped into three categories: healthy (negative controls, in green), moderately affected (orange) and severely affected (red). Bars: standard error of the mean.

Severity was scored from 0 to 3, with 0 being non-affected and 3 given to areas with the maximum lesion intensity. After scoring the lesions, the ten sick animals were further classified into two groups: moderately affected (n = 5) and severely affected (n = 5) groups (Supplementary File S8 and Figure 2).

The group classified as healthy did not show vacuolization in any of the sections or other relevant histological changes of the neuroparenchyma. Very occasionally (in two of the cases selected), discrete and dispersed vacuoles were seen in the internal capsule and medulla oblongata.

In cases showing emaciation and classified as moderately affected, there was a general and consistent bilateral and symmetric neuropil vacuolization found in the transition between grey and white matter, mostly in cortical areas of frontal, parietal, occipital and temporal lobes. Thalamic nuclei, the mesencephalon and the internal capsule at the level of the basal nuclei (but involving only white matter) were also affected. The hypothalamus, hippocampus, pons, medulla oblonga and cerebellum were minimally or not affected.

In wasted animals that showed intense neuropil vacuolization, the cerebral cortex in all sections assessed was intensely affected, but the most altered structures were the thalamic nuclei, particularly ventrolateral ones (reticular nuclei and geniculate body), while dorsomedial thalamic nuclei were more preserved. The mesencephalon’s colliculi and tegmentum were also strongly involved. Vacuoles were numerous and tended to be coalescent in some sections or to line up intermingled between fibers. The internal capsule of the corpus striatum was affected (white matter only as previously mentioned in moderately affected animals, but not the basal nuclei grey matter). Pons and medulla oblongata were slightly affected in the most severe cases. Cerebellar affectation was seen including the cerebellar cortex and nuclei, mostly the interpositus nucleus. Vacuoles were mostly restricted to the cortical granular layer and cerebellar nuclei. Fastigius and dentatus nuclei were only affected in the most severe cases as well. The white matter of the cerebellar medulla was preserved.

**Figure 3:**
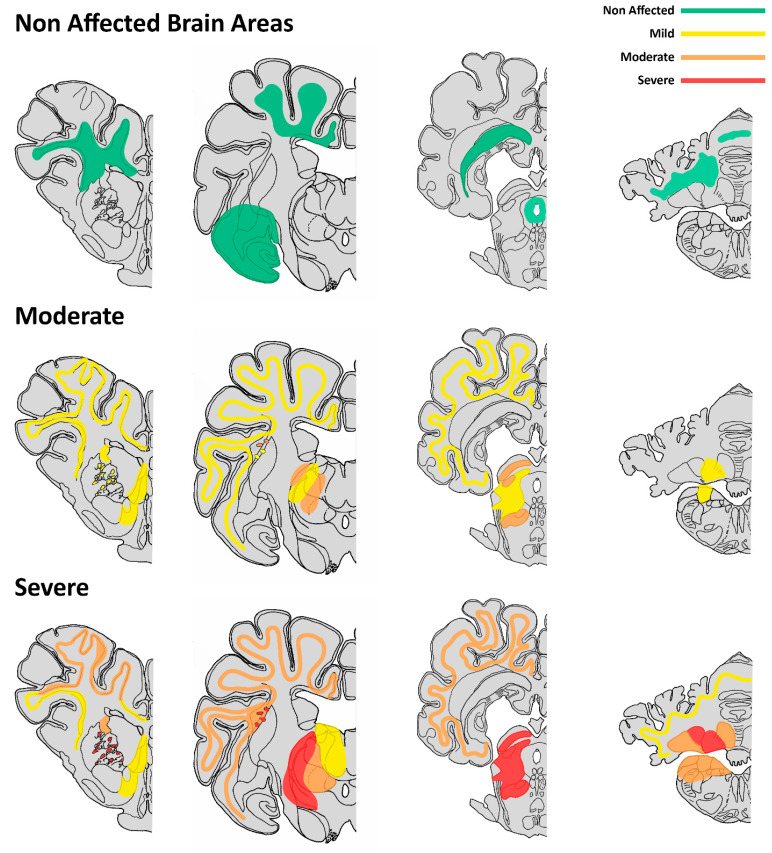
Schematic representation of the intensity of spongiotic lesions in 4 representative brain sections. Top row: the areas that did not show spongiosis in any of the groups are depicted in green. Middle and bottom rows: a summary of the spongiform lesions observed in animals classified into moderately and intensely affected ones is presented, respectively. Areas with scores 1, 2 and 3 are colored in yellow, orange and red, corresponding to mild, moderate and severe affectation, respectively. Graphics based on *Stereotaxic Atlas of the Pig Brain* [19].

In all cases, from the non-affected to the most severe ones, several structures were not damaged, including the corona radiata, corpus callosum, paleocortex (piriform lobule), amygdala, hypothalamus, hippocampus, periaqueductal grey matter, white matter of the cerebellar medulla, dorsal nuclei of the medulla oblongata and spinal cord. A representative brain mapping of lesions in each of the two wasted groups is summarized schematically in Figure 3, based on a pig brain atlas from veterinary literature [19].

Very occasionally, and only in severe cases and in most severe areas, small perivascular cuffs were seen, but no malacic or significant inflammatory changes affecting neuroparenchyma or adjacent tissues such as meninges, choroidal plexuses and ventricles were observed.

### 3.6. Histochemical and Inmunohistochemical Findings of the Brain

**Figure 4:**
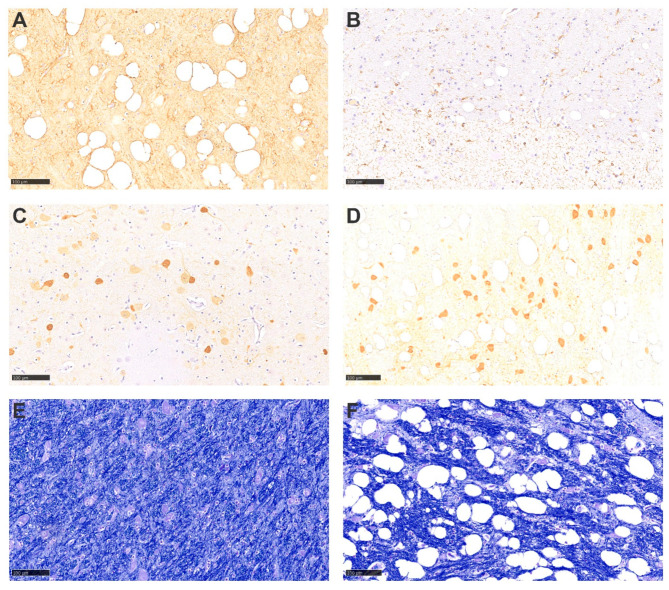
Special stains and immunohistochemistry. (**A**) GFAP antigen detection in an area with marked vacuolation of the parenchyma and absence of gliosis. (**B**) IBA1 antigen detection also in an area with marked vacuolation, with no evidence of gliosis or inflammatory infiltrates. (**C**,**D**) Parvalbumin antigen detection in control and spongiotic cases, respectively, with no evidence of GABAergic inhibitory neuronal population affectation. (**E**,**F**) Luxol fast blue stain of control and spongiotic cases, respectively, with no difference in tinctorial appetence amongst them. All bar scales represent 100 µm.

To characterize the observed microscopic lesions in affected brains, specific immunohistochemical and histochemical studies were performed on the 14 cases selected (Figure 4). Vacuolar lesions were not found to be associated with an evident astroglial or microglial reaction after immunohistochemistry results for GFAP and IBA1 detection. Some vacuoles were surrounded by positive GFAP labeling, but not in a uniform pattern. Immunohistochemistry to determine whether there was a selective involvement of the subpopulation of GABAergic neurons was also performed, with no apparent involvement of the GABAergic parvalbumin+ population, when compared to healthy controls. Moreover, prion protein accumulation was ruled out by means of PrP^res^ immunohistochemistry. In addition, the integrity and morphology of neurons and myelin sheaths were assessed by means of anti-neurofilament immunolabelling pattern and myelin staining with Luxol Fast Blue, but no significant alterations were detected.

### 3.7. Transmission Electron Microscopy 

**Figure 5:**
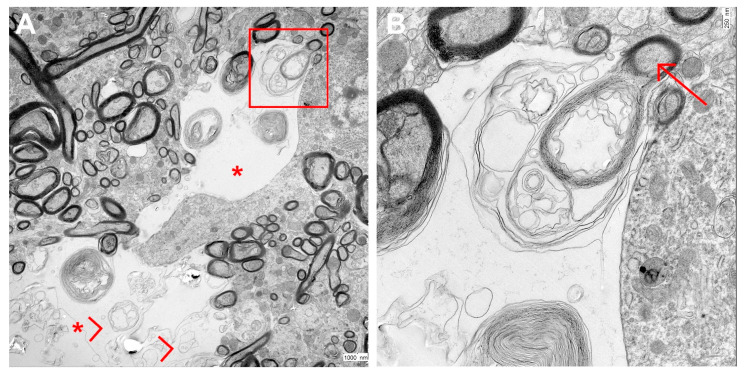
TEM imaging of a lesioned area. (**A**) Dilated structures (*) with thin membranous, partly concentrically arranged structures (arrowhead, most likely representing myelin edema) not enwrapping axonal structures. Bar = 1000 nm. (**B**) Enlargement of the area within the red square. Focal dilation starts within a myelin sheath still enwrapping an axon (arrow). Bar = 250 nm.

To further elucidate the nature of the observed lesions, transmission electron microscopy was conducted in brain samples from two sick animals (with intense lesions) and one healthy animal. Electron microscopy of the brain of the two diseased pigs showed preserved axons, but dilated myelin sheaths revealed marked dilation consistent with vacuolation observed on a light microscopic level. The myelin sheath appeared as loosely arranged lamellae centered around a normally structured axon. Those findings were interpreted as edema of the myelin sheath. No other relevant findings were observed in sick animals. No ultrastructural findings were detected in the healthy animal also examined by transmission electron microscopy (Figure 5).

## 4. Discussion

The present study describes a new condition of swine characterized by forebrain bilateral, symmetrical vacuolization of the neuropil that is clinically manifested by wasting and absence of overt neurological signs. Prevention of the clinicopathological problem was apparently associated with the empirical administration of nutritional vitamin–mineral complexes in drinking water. To the authors’ knowledge, this is the very first description of this type of lesion, which is ultrastructurally characterized by myelin edema and preservation of neurons and axons. It is very likely that the integrity of the neuron and its processes accounts for the lack of clinically detectable neurological signs. It cannot be ruled out, however, that an extensive neurological examination of the animals would have produced more specific data on potential subclinical neurological outcomes. 

Clinically, animals of the wasting group were distinguishable from healthy individuals, showing marked poor body condition and rough coat despite a behavior considered as normal in the herd. Histologically, animals showed bilateral and symmetrical vacuolization of the neuropil. Besides the brain damage, no other remarkable gross or microscopic lesions were relevant or uniformly observed amongst affected individuals. At necropsy, some animals, but mostly the wasted ones, showed lesions that are usually found in intensive swine production systems, such as gastritis, erosion of the *pars oesophagea* of the stomach and focal-to-multifocal pulmonary consolidation compatible with variable degrees of mild suppurative bronchopneumonia [20,21]. However, mild non-suppurative gastritis was most frequently found in sick pigs, likely due to eventual fasting. 

The most striking histopathological finding affecting wasted animals was the presence of bilateral and symmetrical neuropil vacuolization in variable degrees of intensity, which affected specific areas of the brain. The affectation was predominantly at the level of the prosencephalon, mainly located in the thalamic nuclei (but not the hypothalamus) and in the transition between white and grey matter of the neocortex. Thalamic nuclei are mostly related to regulating consciousness, arousal and sleep states, while the neocortex contains a wide variety of regions that are involved in different biological functions depending on the lobe; motor control, perception and cognition as well as other complex behaviors are amongst the most important ones [22]. Other areas were variably affected as well, including the internal capsule and slightly basal nuclei, brainstem, cerebellar nuclei, and in some cases, the granular layer of the cerebellar cortex. Intracytoplasmic vacuoles in the neuropil ultrastructurally corresponded to dilated myelin sheaths, which was interpreted as myelin sheath edema. In the literature, when spongiosis is described mostly in the white matter, congenital disorders, intoxications and metabolic/nutritional alterations are among the most common causes [5]. It is important to highlight that despite those neurological microscopic lesions, none of the sick animals had behavioral or evident neurological alterations suggestive of an intracranial lesion that could be detected by experienced farm veterinarians. It is not possible to rule out whether a more detailed neurological examination would have provided potential evidence of more subtle signs linked to observed lesions. 

Some selected potential infectious causes of the present condition were ruled out by immunohistochemistry (PCV-2, PRRSV, PrP^res^), complementary molecular biology tests (including genome detection of PRRSV, PCV-3 and *Mycoplasma* spp.) or absence of organic lesions compatible with swine influenza or other common swine bacterial or parasitic infectious agents. As no evidence of a common infectious etiological agent and no potential insights about the eventual non-infectious causes for the changes described were found, some management and dietary changes were implemented, with the introduction of vitamin, mineral and hepatoprotective complexes (Supplementary File S1). The use of this product in a curative manner did not impact the outcome of the disease, but the preventive use of it around weaning ameliorated the condition, to the point that its clinical incidence decreased over time, and it disappeared by the end of 2018. Since then, no similar outbreaks have occurred, even after discontinuing the use of the product. The eventual removal of the product at the time this condition was present in the farms implied observing the clinical signs again, further suggesting an efficient outcome of the used vitamin–mineral complex. Altogether, in the absence of an infectious component, the collected evidence is suggestive of a potential vitamin–mineral deficiency that was counteracted by its exogenous preventive administration. 

Metabolic disturbances are mostly inborn (congenital) and rare in animals; they are suspected to be related to mutations in enzymes, which alter the metabolism of specific constituents (amino acids, carbohydrates) or within a specific organelle (mitochondrial enzymes, peroxisome enzymes). In the veterinary literature, cases of brain vacuolization with a congenital basis have been described mostly in dogs, with cases of canine leukoencephalopathy and spongy degeneration with a hereditary basis linked to gene mutations or unknown etiology [23,24,25,26,27]. Bovine Maple Syrup Urine disease is a lethal and autosomal recessive condition in calves caused by a branched chain ketoacid dehydrogenase complex deficiency which leads to the formation of large vacuoles in mainly myelinated white matter tracts of the cerebellum and cerebral cortex, and sites of myelinated fiber terminals, including the brainstem nuclei and spinal cord grey matter [28]. Other cases of unknown origin or with a probably metabolic/genetic basis have been described in the veterinary literature. One of them included a ferret showing neurological signs, with no evidence of etiological agents involved and with a primary neuronal degeneration clinically suspected. Vacuoles were observed mostly in the rostral medulla and cerebellar roof nuclei, but a few were observed in the hippocampus as well [29]. In another case, an 8-month-old cat with vacuolation only of the grey matter of the brain affecting the brain and the spinal cord was also reported. No infectious causes were identified, and thus, a possible congenital degeneration was clinically suspected [30]. In all the cases described above, animals showed neurological signs reflecting cerebellar dysfunction such as ataxia, weakness and reduced spinal reflexes as well as other inflammatory findings affecting the brain, amongst other histological lesions and clinical signs. In the cases of our study, few brains were examined in neonatal pigs coming from the affected farms, but none displayed a neurological lesion. Moreover, a higher number of neonatal brains from other affected farms were examined, and again, no lesions were observed. Therefore, it is suggested that no congenital or perinatal causes were involved in the condition described here.

Vacuolation of the neuropil has been described in dogs intoxicated by the neurotoxic rodenticide bromethalin, with hemorrhages and malacia also observed [31]. A case of a cow with vacuolation at the dorsal vagus nuclei in the medulla oblongata due to lead poisoning amongst other organic findings has also been described [32]. No evidence of intoxication has been proven in the cases described in the present study.

Regarding nutritional causes, neuropil vacuolization has been reported in cats suffering from starvation, hepatic lipidosis and thiamine deficiency, in which the caudal colliculus was the most affected structure [33,34,35]. Malacia, hemorrhages and gliosis are amongst other histological changes described in animals with thiamine deficiency, while in our cases, no inflammatory changes were seen. Reversibility has been proven in those cases with the administration of nutritional additives, as it has apparently occurred with the swine cases described. 

## 5. Conclusions

In summary, the present study describes a new condition of pigs characterized pathologically by brain vacuolization and clinically by wasting with no neurological signs. No underlying infectious or congenital processes were detected. A metabolic disorder was postulated as the potential cause of the disease since the preventive use of vitamin and mineral complexes ameliorated the clinical outcome. In any case, the specific deficiency responsible for this condition was not elucidated. To the authors’ knowledge, this is the first time that the presented findings have been described in swine, which enhances the importance of management and diet composition of rations in swine production, and how dietary imbalances can probably affect the integrity of the brain parenchyma and, consequently, the body condition of the animal. 

## Figures and Tables

**Table 1:** General gross and histological findings of the necropsied animals.

Farm ID (Number of Studied Pigs)	Gross Lesions		Histological Features	
	Healthy	Clinically Affected	Healthy	Clinically Affected
**1** (n = 10)	- Gastric erosions of *pars oesophagea* (1/5).	- Gastric erosions (2/5). - Gastric ulceration of *pars oesophagea* (1/5). - Serous fat atrophy (2/5). - Nasal turbinate atrophy (2/5). - Cranioventral pulmonary consolidation (1/5).	- Non-suppurative rhinitis (4/5). - Inclusion body rhinitis (1/5). - Non-suppurative gastritis (1/5). - Non-suppurative colitis (1/5). - Vacuolization of the thalamus neuropil (1/5).	- Non-suppurative rhinitis (5/5). - Non-suppurative gastritis (4/5). - Ulcerative gastritis (1/5). - Non-suppurative colitis (2/5). - Balantidium coli infection (1/5). - Vacuolation of the thalamus neuropil (3/5).
	Average weight: 16.24 kg. (Range: 14.82–17.56 kg)	Average weight: 12.40 kg. (Range: 11.78–14.18 kg)		
**2** (n = 10)	- Hyperkeratosis of the gastric *pars oesophagea* (3/5).	- No gross lesions.	- Non-suppurative rhinitis (5/5). - Inclusion body rhinitis (2/5). - Non-suppurative gastritis (4/5).	- Non-suppurative rhinitis (4/5). - Inclusion body rhinitis (1/5). - Non-suppurative gastritis (2/5). - Atrophy and fusion of intestinal villi (1/5). - Non-suppurative colitis (1/5). - Vacuolation of the thalamus neuropil (4/5).
	Average weight: 15.59 kg (Range: 12.30–18.34 kg)	Average weight: 9.02 kg. (Range: 8.28–9.42 kg)		
**3** (n = 10) *	Not available.	- Mild cranioventral pulmonary consolidation and focal fibrous pleuritis (1/10). - Gastric erosions of *pars oesophagea* (1/10) - Pulmonary abscess (1/10).	Not available.	- Suppurative bronchopneumonia and pulmonary sequestrum (2/10). - Mild interstitial pneumonia (2/10). - Non-suppurative rhinitis (7/10). - Ulcerative gastritis (2/10). - Non-suppurative colitis (3/10). - Vacuolation of thalamus, cerebellum and pons neuropil (8/10).
		Average weight: 7.4 kg. (Range: 6.20–10.04 kg)		
**4** (n = 10)	- Umbilical abscess (1/5). - Cranioventral pulmonary consolidation (1/5). - Fibrous pericarditis (2/5). - Gastric erosions of *pars oesophagea* (3/5).	- Otohematoma (1/5). - Cranioventral pulmonary consolidation (1/5). - Gastric ulceration of *pars oesophagea* (1/5).	- Non-suppurative rhinitis (4/5). - Non-suppurative colitis (1/5). - Fibrous/fibrinous pericarditis (1/5).	- Non-suppurative rhinitis (4/5). - Non-suppurative gastritis (1/5). - Vacuolization of thalamus, cerebellum and subcortical neuropil (4/5).
	Average weight: 18.04 kg. (Range: 14.20–20.94 kg)	Average weight: 8.88 kg. (Range: 7.46–10.82 kg)		
**5** (n = 11)	- Hyperkeratosis of gastric pars esophagica (2/5). - Gastric erosion of *pars oesophagea* (1/5). - Umbilical abscess (1/5).	- Hyperkeratosis of gastric *pars oesophagea* (2/6). - Erosion/ulceration of gastric *pars oesophagea* (3/6). - Umbilical abscess (1/6). - Pneumonia (1/6).	- Interstitial pneumonia (1/5). - Non-suppurative rhinitis (4/5).	- Suppurative bronchopneumonia (1/6). - Non-suppurative rhinitis (5/6). - Non-suppurative gastritis (5/6). - Vacuolation of the thalamus neuropil (4/6).
	Average weight: 17.02 kg. (Range: 15.00–23.00 kg)	Average weight: 8.2 kg. (Range: 4.50–11.50 kg)		
**6** (n = 10)	Not available.	Not available.	- Vacuolization of the thalamus neuropil (2/5).	- Vacuolization of the thalamus neuropil (4/5).
**7** (n = 5)	Not available.	Not available.		- Vacuolization of the thalamus neuropil (5/5).
**8** (n = 10) First batch	- No gross lesions.	- Variable colonic lymphoid hyperplasia (4/5). - Periarticular abscess (1/5).	- Non-suppurative rhinitis (1/5). - Non-suppurative gastritis (1/5). - Non-suppurative colitis (1/5). -Vacuolization of the thalamus neuropil (1/5).	- Interstitial pneumonia (4/5). - Non-suppurative rhinitis (1/5). - Non-suppurative gastritis (2/5). - Non-suppurative colitis (4/5). - Non-suppurative hepatitis (1/5). - Vacuolization of the thalamus neuropil (4/5).
	Average weight: 14.33 kg. (Range: 9.98–16.70 kg)	Average weight: 8.88 kg. (Range: 7.64–9.58 kg)		
**8** (n = 5) Second batch **	- Not available.	- Not available.	- Not available.	- Vacuolization of thalamus and subcortical white matter neuropil (4/5).

* Based on the clinical condition and weight, all animals selected as healthy by the farmer were considered as sick. ** Five brains of neonates were also additionally included with no significant histological lesions.

## Data Availability

All data generated or analyzed during this study are included in this published article, as well as in the Supplementary Files.

## Supplementary Materials

The following supporting information can be downloaded at: https://www.mdpi.com/article/10.3390/ani13142255/s1, Supplementary File S1: Multivitamin and mineral complex composition. Supplementary File S2: Animal weights. Supplementary File S3: Animals submitted for necropsy and tests performed. Supplementary File S4: Studied biochemical parameters in serum of pigs from farms 1 and 2. Supplementary File S5: Hematology parameters from pigs from farms 1 and 2. Supplementary File S6: Heavy metal determination. Supplementary File S7: Details of antibodies, their dilution and incubation period for the immunohistochemical techniques used in the study. Supplementary File S8: Spongiosis score in different areas of the brain of the 14 selected animals for specific neuropathological studies.
