# Supplementary material for: Porcine Forebrain Vacuolization Associated with Wasting in Pigs: A Novel Pathological Outcome Associated with Vitamin–Mineral Deficiency?
Source: Animals (Basel). 2023 Jul 10;13(14):2255. doi: 10.3390/ani13142255 (PMC10376092; doi:10.3390/ani13142255)
Supplement: Supplementary file 1 [file animals-13-02255-s001.zip › Supplementary file S1.pdf]

Supplementary file S1. Multivitamin and mineral complex composition.

Water-soluble complementary feed in oral solution*

**Composition**	**Per litre**
Dextrose	0.50%
Sodium chloride	0.50%

**Additives**	**Grams**
Trace elements or compounds of trace elements	
Zinc (E6) (glycine chelate hydrate)	15g
Copper (E4) (glycine chelate hydrate)	2.5g
Manganese (E5) (glycine chelate-hydrate)	10g

*0.5 litres of product for every 1.000 liters of drinking water, for 3 weeks, or 1 litre of product for every 1.000 litres of drinking water, for 1 week.

Water-soluble complementary feed in oral solution. Growth stimulant and hepatoprotector**

**Composition**	**Per liter**
Magnesium sulfate heptahydrate	4%
Propylene glycol	2.70%

**Additives per liter**	**Grams**
Sorbitol (E420)	140
Choline Chloride (3a890)	125
D-Panthenol (3a842)	10
Sodium ascorbate (1b301)	6.8
Niacinamide (Vit. B3) (3a315	5
Pyridoxine hydrochloride (Vit. B6) (3a831)	5
Potassium sorbate (E 202).	2.7
Artichoke extract	6
Vitamin E (3a700)	2
Thiamine hydrochloride (Vit. B1) (3a820)	2
Riboflavin phosphate sodium (Vit. B2)	1.4
Cyanocobalamin (Vit. B12)	3 (mg)

**2-3 L for every 1.000 L of drinking water for 7-10 days.
