# Supplementary material for: Porcine Forebrain Vacuolization Associated with Wasting in Pigs: A Novel Pathological Outcome Associated with Vitamin–Mineral Deficiency?
Source: Animals (Basel). 2023 Jul 10;13(14):2255. doi: 10.3390/ani13142255 (PMC10376092; doi:10.3390/ani13142255)
Supplement: Supplementary file 1 [file animals-13-02255-s001.zip › Supplementary file S2.pdf]

Supplementary file S2. Animal weights.

	Reference	Condition	Sex	Weight (Kg)
**Farm 1**	N-364/17	Normal	Female	16.78
	N-365/17	Normal	Female	17.56
	N-366/17	Normal	Male	14.82
	N-367/17	Normal	Male	16.54
	N-368/17	Normal	Male	15.5
	N-369/17	Wasted	Female	11.86
	N-370/17	Wasted	Female	11.78
	N-371/17	Wasted	Male	14.18
	N-372/17	Wasted	Female	12.12
	N-373/17	Wasted	Male	12.04
**Farm 2**	N-354/17	Normal	Male	16.7
	N-355/17	Normal	Female	11.3
	N-356/17	Normal	Male	18.34
	N-357/17	Normal	Male	18.02
	N-358/17	Normal	Female	13.6
	N-359/17	Wasted	Male	9
	N-360/17	Wasted	Male	8.28
	N-361/17	Wasted	Male	8.94
	N-362/17	Wasted	Male	9.42
	N-363/17	Wasted	Male	9.48
**Farm 3**	N-403/17	Wasted	Female	7.64
	N-404/17	Wasted	Male	6.2
	N-405/17	Wasted	Male	7.28
	N-406/17	Wasted	Male	9.1
	N-407/17	Wasted	Male	6.78
	N-408/17	Normal*	Female	8.82
	N-409/17	Normal*	Male	10.04
	N-410/17	Normal*	Female	9.78
	N-411/17	Normal*	Male	10.1
	N-412/17	Normal*	Male	9.24
**Farm 4**	N-436/17	Wasted	Male	9.04
	N-437/17	Wasted	Female	10.82
	N-438/17	Wasted	Male	8.98
	N-439/17	Wasted	Male	8.14
	N-440/17	Wasted	Female	7.46
	N-441/17	Normal	Female	18.6
	N-442/17	Normal	Female	14.2
	N-443/17	Normal	Female	17.84
	N-444/17	Normal	Female	18.6
	N-445/17	Normal	Female	20.94
**Farm 5**	N-510/17	Normal	Male	23
	N-511/17	Normal	Female	16

	N-512/17	Normal	Male	15
	N-513/17	Normal	Female	15.5
	N-514/17	Normal	Male	16.5
	N-515/17	Wasted	Male	8
	N-516/17	Wasted	Male	11.5
	N-517/17	Wasted	Female	9
	N-518/17	Wasted	Male	9.5
	N-519/17	Wasted	Male	4.5
	N-520/17	Wasted	Female	6.5
**Farm 8 (first batch)**	N-392/17	Wasted	Male	9.34
	N-393/17	Wasted	Male	9.68
	N-394/17	Wasted	Male	7.64
	N-395/17	Wasted	Male	8.42
	N-396/17	Wasted	Female	9.36
	N-397/17	Normal	Female	16.7
	N-398/17	Normal	Female	16.58
	N-399/17	Normal	Male	9.98
	N-400/17	Normal	Female	13.96
	N-401/17	Normal	Male	14.42

*Based on the body condition of submitted animals, they were finally classified as also affected by the wasting condition.
