# Supplementary material for: Porcine Forebrain Vacuolization Associated with Wasting in Pigs: A Novel Pathological Outcome Associated with Vitamin–Mineral Deficiency?
Source: Animals (Basel). 2023 Jul 10;13(14):2255. doi: 10.3390/ani13142255 (PMC10376092; doi:10.3390/ani13142255)
Supplement: Supplementary file 1 [file animals-13-02255-s001.zip › Supplementary file S3.pdf]

Supplementary file S3. Animals submitted for necropsy and tests performed.

Farm		Samples submitted	Healthy pigs (n)	Sick pigs (acute) (n)*	Biochemistry/Hematology	Heavy Metals	Molecular biology
**1**		Whole pig	5	5	All pigs	All pigs	PCR (serum) *Mycoplasma* spp. PRRSV, and PCV-3. IHC PCV-2 and PRRSV (lymphoid tissues)
**2**		Whole pig	5	5	All pigs	All pigs	PCR (serum) *Mycoplasma* spp. PRRSV, and PCV-3. IHC PCV-2 and PRRSV (lymphoid tissues)
**3**		Whole pig	NA	10	NP	All pigs	PCR (serum) *Mycoplasma* spp. PRRSV, and PCV-3. IHC PCV-2 and PRRSV (lymphoid tissues)
**4**		Whole pig	5	5	NP	All pigs	PCR (serum) *Mycoplasma* spp. PRRSV, and PCV-3. IHC PCV-2 and PRRSV (lymphoid tissues)

**5**		Whole pig	5	6	NP	NP	PCR (serum) *Mycoplasma* spp. PRRSV, and PCV-3. IHC PCV-2 and PRRSV (lymphoid tissues)
**6**		Brain	5	5	NA	NA	PCR (serum) *Mycoplasma* spp. PRRSV, and PCV-3.
**7**		Brain	NA	5	NA	NA	PCR (serum) *Mycoplasma* spp. PRRSV, and PCV-3.
**8 (first batch)**		Whole pig	5	5	NP	NP	RT-PCR (serum) *Mycoplasma* spp. PRRSV, and PCV-3. IHC PCV-2 and PRRSV (lymphoid tissues)
**8 (second batch)****		Brain	NA	5	NA	NA	PCR (serum) *Mycoplasma* spp. PRRSV, and PCV-3.

*At the peak of the clinical problem.

**Brain of 5 neonates were also submitted for histopathological assessment.

NP = not performed; NA = non-available
