# Supplementary material for: Porcine Forebrain Vacuolization Associated with Wasting in Pigs: A Novel Pathological Outcome Associated with Vitamin–Mineral Deficiency?
Source: Animals (Basel). 2023 Jul 10;13(14):2255. doi: 10.3390/ani13142255 (PMC10376092; doi:10.3390/ani13142255)
Supplement: Supplementary file 1 [file animals-13-02255-s001.zip › Supplementary file S4.pdf]

Supplementary file S4. Studied biochemical parameters in serum of pigs from farms No. 1 and 2.

	**Animal reference**	**Alkaline Phospatase (U/L)**	**Iron (µg/dL)**	**Calcium (mg/dL)**	**Phosphorus (mg/dL)**	**Magnesium (mg/dL)**
**Farm 1**	N-364/17	199.1	93.4	9.5	9.85	2.42
	N-365/17	139.05	70.2	9.4	9.68	2.27
	N-366/17	265.96	39.3	10	11.35	2.63
	N-367/17	248.81	39.9	10.3	10.59	2.37
	N-368/17	359.47	41.4	10.2	11.5	1.99
	N-369/17	331.61	60.6	10.4	10.6	2.59
	N-370/17	329.36	55.9	10	9.47	2.15
	N-371/17	289.63	22	10.3	13.02	2.25
	N-372/17	392.27	81.2	10.3	11.17	2.33
	N-373/17	170.75	6	9.5	8.75	2.15
**Farm 2**	N-354/17	364.89	147.3	10.6	9.23	2.72
	N-355/17	446.23	48.9	9	8.43	2.16
	N-356/17	371.22	65.1	9.9	10.21	2.45
	N-357/17	289.99	64	10.5	11.1	2.74
	N-358/17	337.51	36.8	9.9	10.2	2.37
	N-359/17	231.28	101	9.9	6.44	2.3
	N-360/17	201.41	86.8	10.5	7.66	2.39
	N-361/17	139.11	98.5	10.1	9.43	2.37
	N-362/17	170.02	44.7	9.1	9.66	2.15
	N-363/17	98.1	80.7	9.3	7.41	2.13
Reference values*		120-400	73-140	7.1-11.6	5.3-9.6	1.1-3.7

*Reference values: Clinical Examination of Farm Animals, Peter G.G. Jackson, Peter D. Cockcroft, Blackwell Science Ltd, 2002
