# Supplementary material for: Porcine Forebrain Vacuolization Associated with Wasting in Pigs: A Novel Pathological Outcome Associated with Vitamin–Mineral Deficiency?
Source: Animals (Basel). 2023 Jul 10;13(14):2255. doi: 10.3390/ani13142255 (PMC10376092; doi:10.3390/ani13142255)
Supplement: Supplementary file 1 [file animals-13-02255-s001.zip › Supplementary file S5.pdf]

Supplementary file S5. Haematology parameters from pigs from farms 1 and 2*.

	Animal reference	RBC (x10^6 cel/µL)	WBC (x10^3 cel/µL)	HB (g/dL)	Ht (%)	Neu (%)	Lin (%)	Mon (%)	Eos (%)	Pla (x10^3 plt/µL)	PT (mg/dL)
**Farm 1**	N-364/17	6.96	10.63	10.3	29.4	40.2	56.6	1.8	0.5	661	5.2
	N-365/17	6.74	15.87	9.4	27.9	57	40	1.2	1	944	5.1
	N-366/17	6.18	17.54	9.4	27.6	63.9	32.4	1.6	0.9	819	5.1
	N-367/17	6.87	17.25	9.7	28.2	55.9	40	2.2	0.7	772	5.6
	N-368/17	6.25	17.73	9.5	28.1	69	27.3	2.6	0.8	574	5
	N-369/17	6.54	10.62	9.9	29.7	52.4	44.1	1.9	0.7	487	5.6
	N-370/17	6.24	19.22	9.2	26.6	69.1	27.5	2	0.8	755	5.1
	N-371/17	6.09	12.75	10.1	30.3	58.1	37.5	2.8	0.3	627	5.1
	N-372/17	7.21	24.03	10.8	32.1	77.8	19.9	1.4	0.4	705	5
	N-373/17	3.76	19.54	5.6	17.7	73.4	21.1	4.8	0.4	1079	4.6
**Farm 2**	N-354/17	6.3	18.3	9.9	30	62.3	34.8	1.3	0.4	616	5.4
	N-355/17	4.91	9.79	8.3	25.1	44.7	49.1	2.7	1.4	458	4.4
	N-356/17	5.43	15.62	8.4	24.8	61.9	34.8	1	1.2	588	5.4
	N-357/17	6.15	17.03	9.9	30	58.8	36	3.1	0.8	662	5.4
	N-358/17	5.39	24.5	7.4	23	58.3	35.8	2.6	0.3	649	5
	N-359/17	5.47	15.05	9.7	27.2	57.8	38.2	1.7	0.9	562	5.1
	N-360/17	5.49	18.35	9.5	28.1	68.8	27.1	2.3	0.6	968	5.4
	N-361/17	5.1	13.18	8.2	23.8	51.9	45.4	1.4	0.5	900	5.3
	N-362/17	5.59	16.72	8.4	25	56	41.2	1.2	0.9	500	4.2
	N-363/17	5.29	8.23	9.5	27.3	42.9	51.6	3.5	0.5	516	4.8
	Ref.Values*	5-8	11-22	9-14	26-41	16-73	12-70	0-17	0-6	100-900	4.4-7.4

*Reference values: Cardiovascular system. hematology and clinical chemistry. Diseases of Swine. Iowa State University. 2019.

RBC=red blood cells; WBC=white blood cells; HB=hemoglobin; Ht=hematocrit; Neu=neutrophils; Lin=lymphocytes; Mon=monocytes; Eos=eosinophils; Pla=platelets; PT=total protein.
