# Supplementary material for: Porcine Forebrain Vacuolization Associated with Wasting in Pigs: A Novel Pathological Outcome Associated with Vitamin–Mineral Deficiency?
Source: Animals (Basel). 2023 Jul 10;13(14):2255. doi: 10.3390/ani13142255 (PMC10376092; doi:10.3390/ani13142255)
Supplement: Supplementary file 1 [file animals-13-02255-s001.zip › Supplementary file S7.pdf]

Supplementary file S7. Details of antibodies, their dilution and incubation period for the immunohistochemical techniques used in the study.

**Primary antibody**	**Dilution**	**Manufacturer reference**	**Incubation period**	**Positive control**
GFAP	1:500	DAKO Z0334	ON 4ºC	Brain
NF200 KDa (total)	1:5000	SIGMA ALDRICH N-0142	40' RT	Brain
Iba1	1:300	ABCAM Ab 5076	ON 4ºC	Brain
Parvalbumin	1:500	SIGMA P3088 Clone PARV-19	ON 4ºC	Brain
PRRSV	1:1000	Ingenasa 1AC7	ON 4ºC	Lung
PCV-2	1:1000	Ingenasa M.11.PCV.I36A9	ON 4ºC	Lymph node
PrP^{Sc}	1:100	Prionics 6H4 (7500996)	60′ RT	Brain

ON=overnight; RT=room temperature
