# Supplementary material for: Porcine Forebrain Vacuolization Associated with Wasting in Pigs: A Novel Pathological Outcome Associated with Vitamin–Mineral Deficiency?
Source: Animals (Basel). 2023 Jul 10;13(14):2255. doi: 10.3390/ani13142255 (PMC10376092; doi:10.3390/ani13142255)
Supplement: Supplementary file 1 [file animals-13-02255-s001.zip › Supplementary file S8.pdf]

Supplementary file S8. Spongiosis score in different areas of the brain of the 14 selected animals for specific neuropathological studies.

	Case	Hippocampus	Parietal cortex	Temporal cortex	Frontal cortex	Occipital cortex	Caudate nuclei	Pyriform lobe	Internal capsule	Putamen/pallidus nucleus	Thalamus	Hypothalamus	Mesencephalon	Medulla oblongata	Cerebellar cortex	Cerebellar nucleus
Non-affected	N-366/17	0,00	0,00	0,00	0,00	0,00	0,00	0,00	0,00	0,00	0,00	0,00	0,00	0,00	0,00	0,00
	N-442/17	0,00	0,00	0,00	0,00	0,00	0,00	0,00	0,00	0,00	0,00	0,00	0,00	0,00	0,00	0,00
	N-355/17	0,00	0,00	0,00	0,00	0,00	0,00	0,00	0,00	0,00	0,00	0,00	1,00	0,00	1,00	1,00
	N-356/17	0,00	0,00	0,00	0,00	0,00	0,00	0,00	1,00	0,00	0,00	0,00	0,00	0,00	0,00	0,00
	Non-affected	0,00	0,00	0,00	0,00	0,00	0,00	0,00	0,25	0,00	0,00	0,00	0,25	0,00	0,25	0,25
	sem	0	0	0	0	0	0	0	0,25	0	0	0	0,25	0	0,25	0,25
Moderate	N-372/17	0,00	2,00	1,00	2,00	1,00	2,00	0,00	1,00	0,00	1,00	0,00	0,00	1,00	0,00	0,00
	N-364/17	0,00	1,00	1,00	1,00	1,00	2,00	0,00	1,00	0,00	2,00	0,00	0,00	0,00	0,00	0,00
	N-359/17	0,00	1,00	1,00	1,00	1,00	1,00	0,00	1,00	0,00	2,00	0,00	0,00	0,00	0,00	0,50
	N-360/17	0,00	0,50	0,50	1,00	1,00	0,00	0,00	1,00	1,00	2,00	0,00	0,00	0,00	0,00	0,50
	N-370/17	0,00	1,00	1,00	1,50	1,00	2,00	0,00	1,50	1,00	2,50	0,00	2,00	1,00	0,00	0,50
	Moderate	0,00	1,10	0,90	1,30	1,00	1,40	0,00	1,10	0,40	1,90	0,00	0,40	0,40	0,00	0,30
	sem	0	0,24494897	0,1	0,2	0	0,4		0,1	0,24494897	0,24494897	0	0,4	0,24494897	0	0,12247449
Intense	N-403/17	0,00	2,00	2,00	2,50	2,50	2,50	0,00	2,00	0,00	3,00	0,00	3,00	0,50	0,50	1,50
	N-438/17	0,00	2,50	2,50	3,00	2,50	2,50	0,00	2,00	1,00	3,00	0,00	3,00	0,50	0,50	1,00
	N-410/17	0,00	3,00	3,00	3,00	2,00	3,00	1,00	3,00	0,00	3,00	0,00	3,00	1,00	1,00	2,50
	N-404/17	0,00	2,50	2,50	2,00	2,00	2,00	1,00	2,50	1,00	3,00	0,00	3,00	1,00	2,00	2,00
	N-361/17	0,00	1,50	2,00	3,00	2,00	1,50	1,00	3,00	1,00	2,00	0,00	2,00	1,00	1,00	1,00
	Intense	0,00	2,30	2,40	2,70	2,20	2,30	0,60	2,50	0,60	2,80	0,00	2,80	0,80	1,00	1,60
	sem	0	0,25495098	0,18708287	0,2	0,12247449	0,254951	0,244949	0,2236068	0,24494897	0,2	0	0,2	0,12247449	0,27386128	0,29154759
